# Developing the research roadmap together: a report from the patient-centered outcome research institute (PCORI) and pediatric surgical oncology research collaborative (PSORC)

**DOI:** 10.1007/s00383-026-06399-2

**Published:** 2026-03-22

**Authors:** Peter F. Ehrlich, Elisabeth T. Tracy, Barrie Rich, Dave R. Lal, Timothy B. Lautz, Richard D. Glick, Erin G. Brown, Janet Panoch, Roshni Dasgupta

**Affiliations:** 1 C.S. Mott Children’s Hospital, University of Michigan, Ann Arbor, MI USA; 2 Pediatric Surgery, Department of Pediatric Surgery, University of North Carolina, Chapel Hill, NC USA; 3 Donald and Barbara Zucker School of Medicine, Hofstra University, Cohen Children’s Medical Center New York, Queens, USA; 4 Division of Pediatric Surgery, Professor of Surgery, Children’s Wisconsin/Medical College of Wisconsin, Milwaukee, USA; 5 Department of Pediatric Surgery, Ann & Robert H. Lurie Children’s Hospital of Chicago, Chicago, USA; 6 Division of Pediatric Surgery, Department of Surgery, University of California Davis Children’s Hospital, Sacramento, CA USA; 7 Indiana University School of Medicine, Indianapolis, USA; 8 Division of Pediatric General and Thoracic Surgery, Division of Pediatric Surgery, Cincinnati Children’s Hospital Medical Center, Cincinnati, OH USA

**Keywords:** Pediatric cancer, Patient centered outcomes research institute, Surgery, Research agenda

## Abstract

**Objective:**

Patient-Centered Outcomes Research (PCOR) and Comparative Effectiveness Research (CER) focusing on pediatric cancer surgery are insufficient. To address this need, the authors aimed to create an evidence-based PCOR agenda for children with solid tumors.

**Methods:**

Between September 2021 and October 2022, the Pediatric Surgical Oncology Research Collaborative (PSORC) advocacy group, composed of 25 physicians and 25 nonmedical patient/parent stakeholders, developed a research agenda culminating in an in-person meeting. Stakeholders included parents of children treated for solid tumors, survivors of childhood cancers, patient advocates, and pediatric oncology (medical and surgical) providers. A multisource five-component framework was used to develop the roadmap: (1) Education, (2) Topic Generation, (3) Gap Analysis and Systematic Review, (4) Value of Information (VOI) Analysis, and (5) Peer Review. Topic generation involved both physician and stakeholder meetings, focus groups, generating a word cloud, and a survey sent to 48 solid tumor disease and advocacy support groups representing over 1000 families. VOI analysis and peer review were conducted in person with 50 participants (25 stakeholders/25 physicians). Descriptive and thematic results are presented.

**Results:**

A systematic review identified only a single surgical PCOR report addressing pediatric solid tumors. Gap analysis demonstrated that surgeons’ goals focused primarily on improving surgical outcomes, whereas stakeholders’ concerns centered on surgeon skill/expertise, second opinions, pain, healing, and communication. The word cloud session identified several key issues: lack of family resources, pain, enhanced recovery, and communication. Important thematic PCOR questions from the survey focused on patient/family lack of knowledge, overwhelming predicaments, immediate surgical treatment options, and shared decision-making between families and surgeons. VOI analysis, peer review, and voting inferred that the primary PCOR agenda should aim to develop a question aid for families/caregivers that increases parent knowledge, engagement, and comfort with cancer surgery and tests the impact of shared decision-making for second opinions to increase parent comfort and participation in the child’s cancer treatment.

**Conclusions:**

Surgical PCOR is lacking and needed to enhance interactions between surgeons and patients/families with pediatric solid tumors. There is a significant discrepancy in the topics and prioritization of PCOR between pediatric surgeons and families. Identifying, understanding, and addressing gaps between patients/families and surgeons may lead to a robust patient-informed research agenda.

## Introduction

 In a 2008 report, the United States Congress identified a need for a medical research agenda addressing questions most relevant to patients and lay caregivers [1]. The goal was to help “people and their [lay] caregivers communicate and make informed healthcare decisions.” The report documented the paucity of patient input on research priorities and significant health disparities in different disease outcomes, such as cancer, between minority and nonminority populations. As a result, in 2010, the Patient-Centered Outcomes Research Institute (PCORI) was established as part of the US Patient Protection and Affordable Care Act [2]. The mission of this institute is to fund patient-centered comparative clinical effectiveness research, extending the concept of patient-centeredness from health care delivery to healthcare research. There are several key aspects to PCOR: (1) to assess the benefits, harms, comparisons, and outcomes of health care interventions to inform decision-making; (2) to include individual preferences, such as health-related quality of life; (3) to include research in diverse settings and populations addressing barriers to implementation and dissemination; and (4) to optimize outcomes from multiple stakeholders, individual burden, and availability of services [3]. A first step is to develop a PCOR agenda that produces an expanded clinical knowledge base relevant to the needs and concerns of patients and caregivers.

Cancer is one of the leading causes of death in children. Each year in the US, 1 out of every 6,000 children develops cancer, and 20,000 children will die from their disease [4, 5]. A significant research gap has been recognized regarding family-centered and comparative effectiveness questions for cancer surgery in children [6, 7]. Traditional chemotherapy-focused studies in pediatric solid tumors have limited opportunities to study the role of surgery and its impact on patient-centered outcomes. Therefore, the Pediatric Surgical Oncology Research Collaborative (PSORC) prioritized this area for research. In 2022, PSORC received a Eugene Washington Engagement Project Award, enabling it to identify specific gaps in pediatric surgical cancer care related to diagnosis, treatment, and outcomes, with the aim of developing patient-centered priorities for future research studies [8]. This project also focused on enabling caregivers of patients with pediatric solid tumors to engage in planning new research on surgical outcomes and comparative effectiveness, develop a patient-centered model for research, and create a roadmap for future research based on patient-centered priorities. This report outlines the methodology and outcomes for creating a PSORC evidence-based PCOR research agenda for children with solid tumors.

## Methods

An IRB exemption was obtained for this research from the University of Michigan. The Pediatric Surgical Oncology Research Collaborative.

PSORC, was established in 2017 [8]. PSROC was originally founded in North America including US programs and a program in Toronto Ontario Canada. Currently there are 55 centers including two in Canada and one in the Netherlands with a few more in Europe working through the membership application. At the time of the meeting and for this publication PSORC had 37 centers just in the USA and Canada. A list of centers can be found at www.psorc.org. Its primary mission is to develop multi-institutional research collaborations to improve surgical outcomes for children and young adults with cancer. PSORC centers have an institutional membership. The leads are pediatric general surgeons but there are several pediatric urologists, pathologist, orthopedic oncologist and pediatric oncologists who participate and propose studies. PSORC has focused on surgical issues not easily studied by traditional cancer consortiums, such as the Children’s Oncology Group. In 2020, PSORC established a stakeholder advisory group to develop and guide research in PCOR.

### Study timeline and framework

**Fig. 1:**
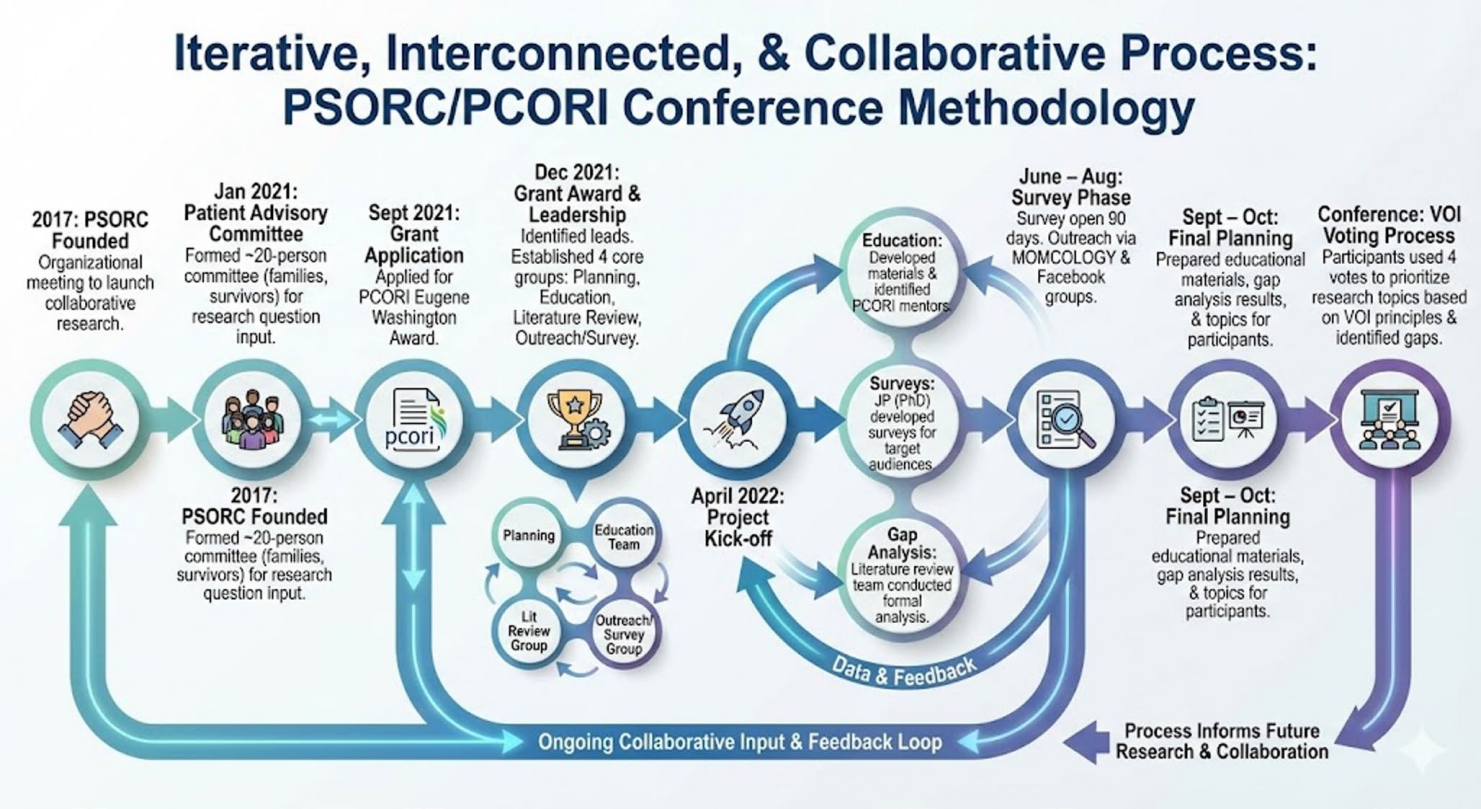
Graphical representation of the iterative process for the study

A detailed timeline for the study conceptualization, development and execution is shown in Table 1. Figure 1 is a graphic representation of the iterative and interactive methodology used for this process. study.

**Table 1:** Study Timeline

Date	Activity	Description
2017	PSORC Founded	Organizational meeting to launch the collaborative research project.
Jan 2021	Patient Advisory Committee	Formed a committee of ~ 20 participants (families and survivors) to develop research questions and provide input for quarterly meetings.
Sept 2021	Grant Application	Applied for the PCORI Eugene Washington Award.
Dec 2021	Grant Award & Leadership	Identified patient, advocate, and physician leads. Established four core groups: Planning, Education, Literature Review, and Outreach/Survey.
April 2022	Project Kick-off	• Education: Developed materials and identified PCORI mentors. • Surveys: JP (PhD) developed surveys for target audiences. • Gap Analysis: Literature review team conducted a formal analysis of research gaps.
June–Aug	Survey Phase	Survey opened for 90 days. Outreach conducted with parent disease-specific organizations (e.g., MOMCOLOGY) and Facebook groups.
Sept–Oct	Final Planning	Preparation and delivery of educational materials, gap analysis results, and potential research topics to all participants prior to the meeting.
Conference	VOI Voting Process	Following topic presentations/discussions, each participant used 4 votes to prioritize research topics based on VOI (Value of Information) principles and identified gaps.

### Framework

**Fig. 2:**
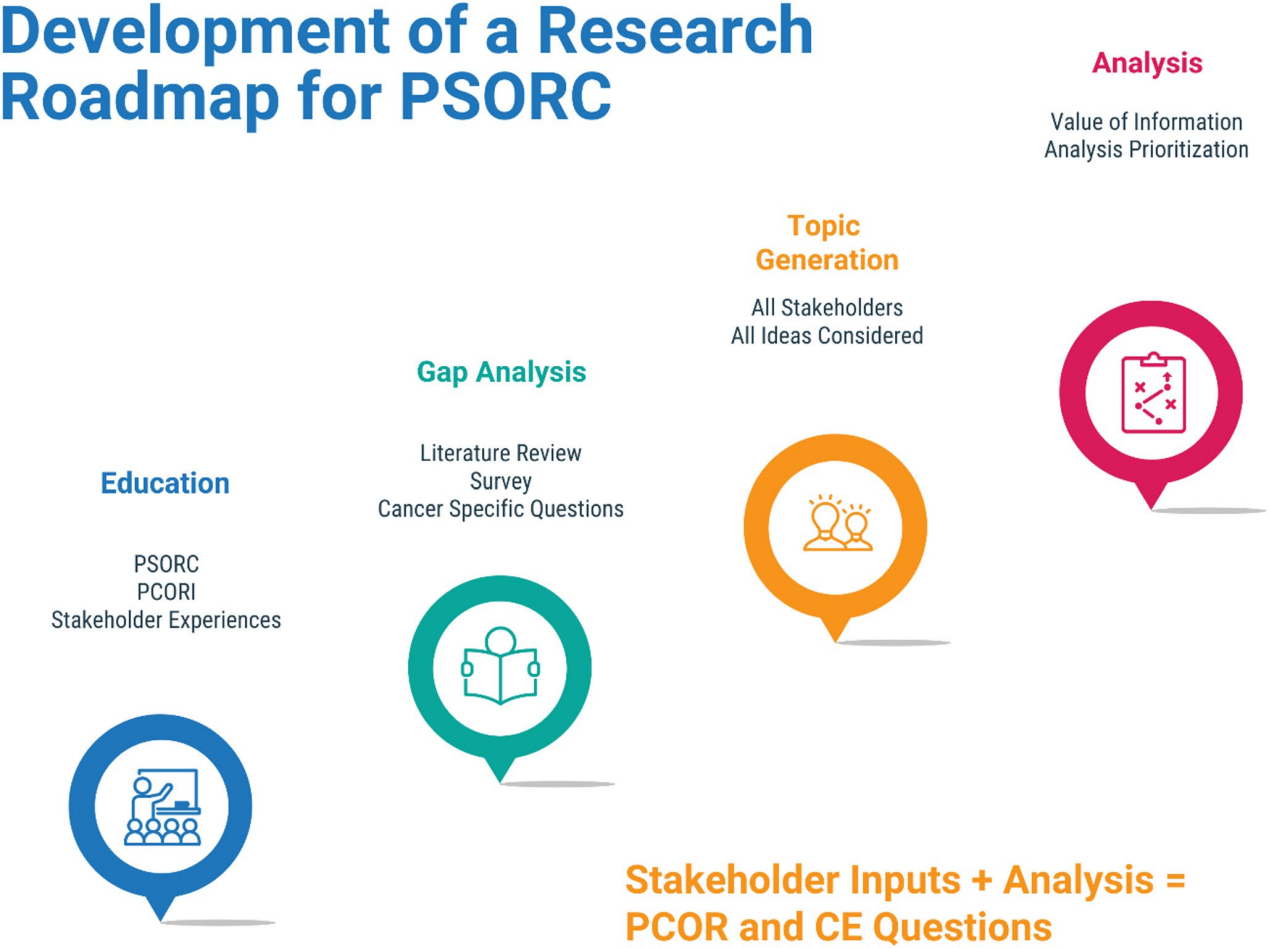
Framework for the research roadmap for PSORC

We adapted a framework developed by PCORI for identifying and prioritizing research areas, focusing on input from parents, childhood cancer survivors, and key stakeholders [9]. A multisource five-component framework was used to develop the roadmap: (1) Education, (2) Topic Generation, (3) Gap Analysis Systematic Review, (4) Value of Information (VOI) Analysis, and (5) Peer/Stakeholder Review. (Fig. 2). Each of these components informed the subsequent steps and the development of the research agenda.

### Education

Education of all stakeholders focused on PCOR/CER and PCORI funding. This education was critical to developing questions that PCORI supports, which are not traditional. For example, PCORI would not fund a trial comparing one chemotherapeutic agent(s) to another but would consider a project comparing limb-sparing surgery to non-limb-sparing surgery and functional outcomes or a study that impacts adherence to treatments. This was particularly important for the medical professionals. Presentations from established and funded PCORI investigators helped elaborate a research roadmap, showcasing how other pediatric investigators had successfully developed extramurally funded research programs.

### Topic generation

Topic generation involved physician and stakeholder meetings, focus groups, and word clouds. We also developed a social media (Facebook) survey, which was sent to 48 solid tumor disease and advocacy support groups. These groups were identified by stakeholders’ knowledge of the groups and a Facebook search using the terms liver tumors, renal tumors, sarcomas, and neuroblastoma. Once the survey was approved by the group’s administrator, it was posted and left up for 30 days [10]. 

### Survey and thematic analysis

The survey consisted of 30 open- and closed-ended questions. A detailed qualitative analysis of the survey is beyond the scope of this publication and will be the subject of a separate report. Thematic analysis was performed on the results of the survey. The survey team created, sent out, and analyzed the survey prior to the conference. Qualitative analysis was used in a systematic fashion to interpret the non-numerical data from the survey. Data was organized, sorted, coded and then grouped into themes for development. Grounded theory was then used to explain the findings. The team was led by author JP, who holds a PhD in decision analysis and has conducted research in thematic and qualitative analysis. The full results of the survey will be published in a future publication. Thematic analysis, primarily used in qualitative research, identifies recurring patterns or themes within a dataset by carefully examining and interpreting the data. For example, two specific open-ended questions were *“Please describe your decision-making experience for the surgery” and “What recommendations do you have for improving the surgical experience before or after surgery?”* This method was used for identifying topics for the research agenda and analyzing the post-conference survey. Descriptive and thematic results are presented.

### Word cloud

A word cloud session is a useful method to visualize the most frequently mentioned terms or topics from a collection of text data, like focus group discussions, open-ended survey responses, or interviews. This identified key themes, which helped to formulate preliminary questions that were eventually worked into structured questions for the value of information analysis and the peer review. This diverse input was used to involve stakeholders in the planning, agenda development, and implementation of the conference.

### Systematic review and gap analysis

The systematic review was done using PubMed, Ovid and the Cochrane library. To be included in the review a paper had to be about a pediatric solid tumor in the pediatric population, a focus on surgery, have patient reported outcomes and or compare different surgical therapies or outcomes form a surgical cancer procedure.

The PubMed search terms used were - ((“patient-centered“[Title/Abstract] OR “patient centered“[Title/Abstract] OR “patient reported“[Title/Abstract] OR “quality of life“[Title/Abstract] OR “patient preferences“[Title/Abstract] OR “patient outcomes“[Title/Abstract] OR “Patient Outcome Assessment“[MeSH] OR “Quality of Life“[MeSH] OR “Patient Satisfaction“[MeSH]) AND (“comparative effectiveness“[Title/Abstract] OR “comparative study“[Title/Abstract] OR “outcomes research“[Title/Abstract] OR “Comparative Effectiveness Research“[MeSH]) AND (“pediatric“[Title/Abstract] OR “child“[Title/Abstract] OR “children“[Title/Abstract] OR “adolescent“[Title/Abstract] OR “Pediatrics“[MeSH] OR “Child“[MeSH] OR “Adolescent“[MeSH]) AND (“surgical oncology“[Title/Abstract] OR “surgery“[Title/Abstract] OR “neoplasm“[Title/Abstract] OR “cancer“[Title/Abstract] OR “tumor“[Title/Abstract] OR “malignancy“[Title/Abstract] OR “Surgical Procedures, Operative“[MeSH] OR “Neoplasms“[MeSH] OR “Oncology“[MeSH]))

The Ovid search terms were.

1. (“patient-centered” or “patient centered” or “patient reported” or “quality of life” or “patient preferences” or “patient outcomes”).mp.
2. Patient Outcome Assessment/.
3. exp Quality of Life/.
4. exp Patient Satisfaction/.
5. 1 OR 2 OR 3 OR 4.
6. (“comparative effectiveness” or “comparative study” or “outcomes research”).mp.
7. exp Comparative Effectiveness Research.
8. 6 OR 7.
9. (pediatric or child or children or adolescent).mp.
10. exp Pediatrics/.
11. exp Child/.
12. exp Adolescent/.
13. 9 OR 10 OR 11 OR 12\.

The Cochrane search terms were.

(“patient-centered” OR “patient centered” OR “patient reported” OR “quality of life” OR “patient preference” OR “patient satisfaction”) AND (“comparative effectiveness” OR “comparative study” OR “outcomes research”) AND (pediatric OR child OR children OR adolescent) AND (“surgical oncology” OR “surgery” OR “neoplasm” OR “cancer” OR “tumor” OR “malignancy”).

### Tumor teams

In preparation for the conference. Surgeons and stakeholder leads were identified for each of the four solid tumors (Renal, Liver, Sarcoma, Neuroblastoma). These included three surgeons who were on the COG solid tumor disease group and three stakeholders (typically and patient advocate, parent and a leader of a survivor group etc.)

### Value of information analysis

Value of Information Analysis is a decision-making tool. VOI analysis quantifies how much uncertainty in decision-making would be reduced if more information (from further research) were available, typically in the context of healthcare interventions or treatments. It helps prioritize research by showing where additional evidence might make the biggest impact on patient outcomes or healthcare costs. VOI analysis doesn’t directly generate research questions, but it identifies where uncertainty is most important to stakeholders. It helps you decide which research study to invest in based on the expected outcomes, patient/population benefit, feasibility, etc. The systematic engagement of multiple stakeholders, especially patients, is a critical component of topic generation. This is followed by a multistep prioritization beginning with systematic reviews and gap analysis to identify what is known, and what desirable knowledge is not known. Although these steps are depicted as linear, in practice, prioritization and reprioritization occur at all stages of the process. The integration of patient viewpoints is included at all stages of research prioritization [11]. 

### Selection of participants

#### Stakeholders

We identified stakeholders through multiple methods. First, we leveraged our association with the Children’s Oncology Group (COG). We contacted the head of the patient advocacy group – COG- and reached out to the patient advocate in each of the four solid organ groups (e.g., Neuroblastoma). The two PIs had prior associations with groups related to Sarcoma (RD) and renal and liver tumors (PFE), as well as organizations like the International WAGR Group. Additionally, we spoke with the leaders of the MOMcology project (momcology.org) and other solid organ tumor advocacy groups. Another method of recruitment was through word of mouth from other families. We had representatives from all four areas in the United States. All expenses for the stakeholders were covered, and those who participated in the conference development and pre-conference work were financially compensated as per PCORI policy.

Physicians. Twenty-two of the physicians’ participants were pediatric surgeons with a prior interest in pediatric oncology, were active in the PSORC consortium, and were interested in PCOR research. Two physicians were emergency medicine doctors with funding from PCORI for pediatric research. The final physician was a pediatric oncologist with a leadership position in a North American pediatric oncology research consortium.

#### Peer stakeholder review

After completing Steps 1–4, a set of questions was created and then underwent peer review and prioritization to form the research agenda. This list of questions was then discussed and refined to ensure clarity and eliminate duplicates. A final list of 10 questions was established. Each of the 50 participants was given four votes to choose the questions they found most interesting.

## Results

### Systematic review

The initial systematic review using general search terms identified 1513 abstracts. Then a second search was performed using the refined search strategy described in the methods. Fifty-one papers met criteria from PubMed, 37 in the IVID search and Pub med search 51 papers and 2 reviews and 25 trials from the Cochrane library. After removing all duplicated abstracts and those that did not meet criteria only one surgical PCOR report addressing pediatric solid tumors [7]. This paper studied scoliosis following thoracic surgery and therapy in children with chest wall sarcomas.

### Conference

The Eugene Washington Engagement Conference took place in Ann Arbor, Michigan, in October 2023, with 50 participants (25 from the medical field and 25 including pediatric cancer survivors, their parents/caregivers, and advocacy group leaders). Table 2 lists the conference agenda.

**Table 2:** Conference agenda

Day 1
A. Setting the stage – why we are here
B. PCORI Basics
C. What makes a successful PCORI grant
D. Solid Tumor Primers and research questions from the surgeon and parent perspective
Renal, Neuroblastoma, Hepatoblastoma and Sarcoma
E. Results for the Survey – Thematic and Qualitative Analysis
F. Bridging our Perspectives on Kids Cancer
Word Cloud
Small Group Discussions
Small Group Presentations
PCOR Research ideas
Day 2
A. Presentation of research questions
B. Discussion and ranking of research ideas
a. Fatal flaws
b. Challenges
c. Voting
C. Summary and Next Steps

To help develop with the generation of PCOR and CER questions the flow from each step was:

1. Describe and educate conference participated about PCORI research funding.
2. Present cancer specific research ideas from a patient and physician perspective.
3. Present the results the qualitative survey.
4. Then small group breakout to develop specific questions.
5. Small group presentations where ideas were refined, merged and that led to a list of 10 ideas which were voted on during the second day of the conference.

**Fig. 3:**
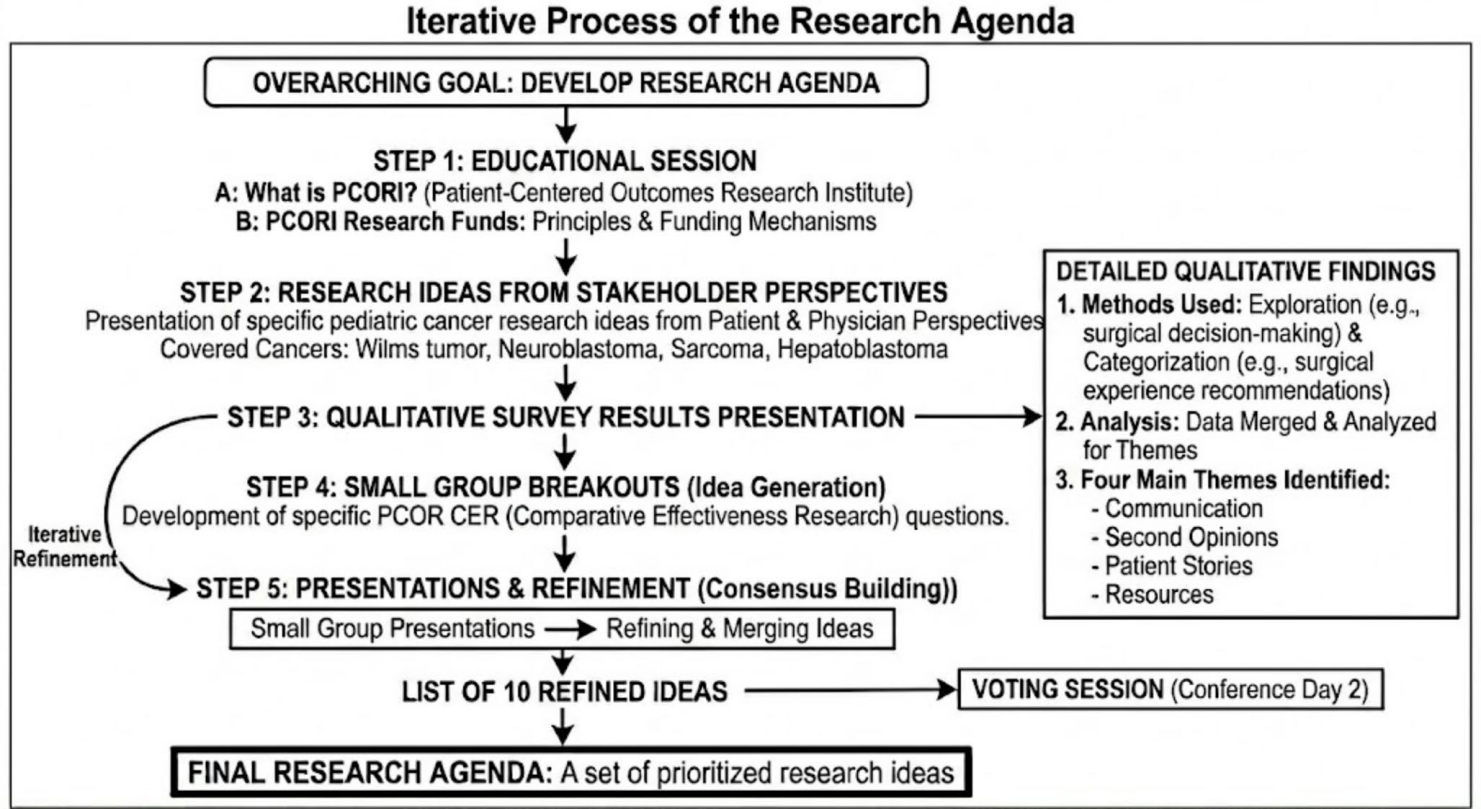
Iterative process of the research agenda

The processes used were iterative and overlapping (Fig. 3).

A critical concept was understanding which studies PCORI might fund and what constitutes a successful PCOR project. To address this three successful PCORI grant recipients who have worked in pediatric fields to advise us. One of them gave a presentation on how to successfully develop and obtain a CER/PCOR grant. The recommendations were derived from this presentation and the mentors’ input. (Table 3)

**Table 3:** Recommendations for a successful PCORI grant

Success	Pitfalls
Pick a real-world problem. Engage patients early and throughout. Use plain language. Must be scientifically rigorous. Compares efficacious/effective intervention. Has high potential for impact scalability. Take the patients perspective. Uses all the relevant stakeholders. Feasible, specific milestones. Have a great team.	Not involving patients or stakeholders. Proposed to develop or validate an instrument. Compares interventions without evidence of efficacy or effectiveness. Proposes a cost effectiveness analysis. Focuses on the cost of care as a primary criterion for choosing between interventions. Specific aims differ between the LOI and the full grant submission.

**Fig. 4:**
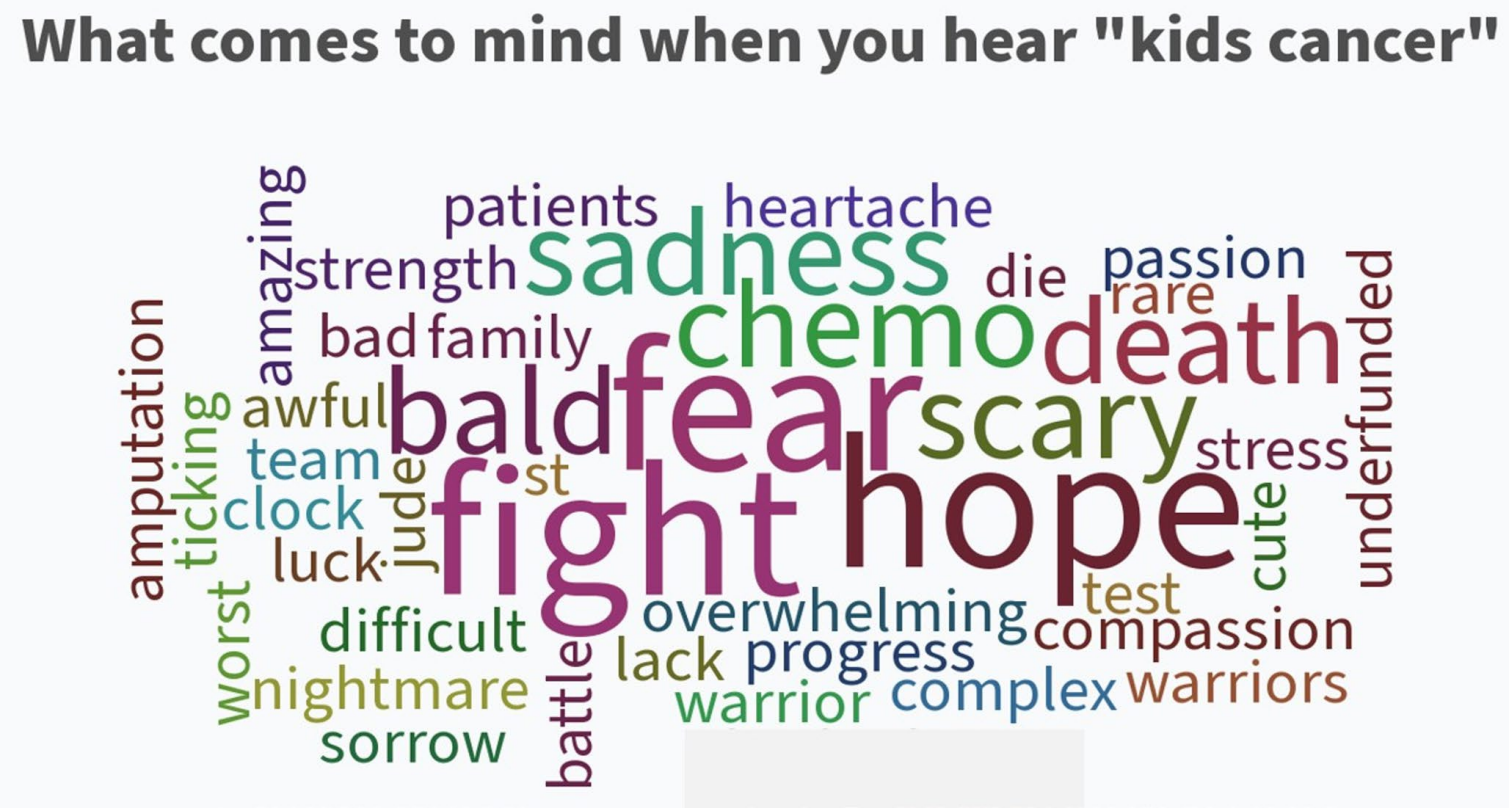
Word cloud-what comes to mind when you hear the words “kids cancer”?

Gap analysis showed surgeons focused primarily on improving surgical outcomes, while stakeholders were concerned with surgeon skill/expertise, second opinions, pain, healing, and communication. (Table 4A-D). The word cloud session allowed non-hierarchical idea generation, emphasizing shared goals and needs, leading into small group discussions. (Fig. 4)

**Table 4:** Tumor specific research from the surgeons and caregiver perspective

Surgeon	Caregiver
**Neuroblastoma**	
What is the right biopsy technique? What preoperative scans are needed (MRI/CT/MIBG/3-D imaging/modeling) to improve surgery outcomes? How to achieve optimal local control in the tumor bed? What is the role of surgery vs. radiation therapy? What is the role of surgery in preparing for subsequent therapies: radiation clips, biopsies of various organs etc…? What are the common causes of an unsuccessful surgery (less than optimal) - margins, complications to other tissues, poor healing or function?	Non-operative treatment of neonates or infants with low-risk disease 1.How do parents feel about leaving in tumor and non-operative management, surveillance imaging and labs? 2. How to best communicate this treatment strategy Optimal therapy for patients with high-risk disease and the role of surgery Improved surgeon to parent/patient communication. Enhanced understanding of operation, pre-and post-op steps, risks, anticipated outcomes Is there a particular problem area that seems most in need of attention and advancements?
**Sarcoma Tumors**	
1.What is the best management of metastatic sites, particularly in the lungs? 2.What is the most effective way to discuss with families and patients (particularly adolescents), when there is a choice about management approach 3.What is the best way for surgeons to engage families in surgical innovation 4.What is the best management of metastatic sites, particularly in the lungs? 5.What is the most effective way to speak with families and patients (particularly adolescents), when there is a choice about management approach? ?	1.How do I know whether my or my child’s surgeon has the skills to ensure the best outcome of clear margins while maximizing future function? 2.How can I understand the experience of survivors living with the two surgery options we are faced with? (example: amputation vs. limb salvage) 3.What can I do to support my or my child’s healing process after surgery? 4.How does parent known that a true multi-disciplinary discussion has occurred, and how does the parent receive feedback from that discussion/participation in that discussion? 5.Is the surgeon willing to discuss a second opinion and explore a different strategy 6.How can I understand the experience of survivors living with the two surgery options we are faced with? (example: amputation vs. limb salvage)
**Renal Tumors**	
Improved therapy for children with high-risk disease Improved therapy for Relapse disease Improved ability to safely spare the kidney.	1.General confidence in surgeon/surgical team 2. Tumor spill 3.Port placement 4.Eating/drinking before and after surgery
**Liver Tumors**	
1.How do various care teams work together to achieve the optimal course of treatment and ultimately, result? 2.How is surgical control of the primary tumor decided? 3.What is the optimal strategy for relapsed disease control?	1.How experienced is my team, are they consulting additional expertise during unique situations? 2.How involved is the tumor, why is it not resectable? 3.Do my surgeons understand the long-term effects of ‘heroic’ resections vs. transplant? Does the team have a bias towards one or the other? 4.Is the surgical team familiar with the latest technologies available, like live imaging, ICG, or new scanning fidelity?

**Table 5:** Research Ideas

Topic
Resources 1. Multimedia “what should parents ask, are you comfortable with the care you are getting 2. Family satisfaction as a primary outcome
Second Opinion 1. Help surgeons with second opinions
Enhanced Recovery protocols (ERAs) 1. Enhanced recovery protocols with family involvement 2. Intervention with a infographic
Communication topics 1. Implications of relapse 2. Socioeconomic studies and how to mitigate disparities 3. Optimal ways to engage parents. 4. Optimizing and empowering parents right from the beginning 5. When they are options how to help parents with best decision for them 6. Healing 7. Participation in tumor boards 8. Intervention to provide information or parent checklist. 9. Development of visual aids
Maximizing research with families 1. Enhancing donation of Tissue Sample 2. Families do not know what they don’t know. 3. Fertility preserving 4. Best practices
Pain 1. Education would involve anesthesia
Long term Outcomes Functional Impact of therapy on life

There were 165 responses to the survey. As this was a social media survey, an estimate of active members for numerators and denominators cannot be accurately determined. After organizing, sorting and coding the survey data the four main themes that emerged were: Communication, Second Opinions, Patient Stories and Resources.

The word cloud session allowed non-hierarchical idea generation, emphasizing shared goals and needs, leading into small group discussions. (Figure X)

These discussions generated 45. additional research ideas, which were workshopped in small group meetings and later presented to all attendees. Family resources, pain, enhanced recovery, and communication were identified as key issues. (Table X) The research ideas were recorded and summarized at the end of day one. Each attendee received a copy of the generated research questions; duplicate questions were combined. This process produced a list of 10 feasible PCOR/CER questions.

When the conference attendees arrived on day two, the 10 questions were written on flip charts and posted around the conference room. Each participant was given four votes to select the questions they felt were most important. A simple majority was used for ranking. The top three questions received 80% or more of all votes, while the fourth question achieved a 70% consensus.

The top four prioritized study issues were.

1. Develop a question aid: “What do I need to ask my surgeon?” for families/caregivers to increase parent knowledge, engagement, and comfort with cancer surgery.
2. Test the impact of shared decision-making for second opinions to enhance parent comfort and participation in the child’s cancer diagnosis.
3. Examine whether parent participation in enhanced recovery protocols reduces hospital length of stay and surgical complications.
4. Develop a method/framework for combined patient/parent/research evaluation of future PSORC research proposals.

Researchers gained a better understanding of the value of patient perspectives in planning research priorities for PSORC. Including patient representatives in planning and designing clinical studies may lead to relevant and important research, generalizable to improve patient outcomes.

## Discussion

Our major finding was the gap between the research priorities of family caregivers and pediatric surgeons. This conclusion emerged from diverse input sources, including literature, patient advocacy groups, a social media survey, and feedback from 25 stakeholders and 25 physician participants. Pediatric surgical oncologists focused on improving survival rates, reducing complications, and enhancing outcomes. In contrast, families prioritized effective decision-making (e.g., “What do I need to ask?”), ensuring their child received the best treatment (such as obtaining second opinions), optimizing pain control, and reducing hospital stays while enhancing inpatient experiences (e.g., through enhanced recovery pathways). These differences likely stem from the fears and overwhelming concerns associated with a cancer diagnosis in their child. We believe seeking second opinions does not signify mistrust in the treating team; rather, parents whose surgeons consulted with other experts felt reassured that their child was receiving the appropriate therapy, thus increasing confidence in their treating team.

Another interesting outcome was group presentations and discussions on strategies and pitfalls for successful PCORI grants. These sessions better equipped us to tailor the research agenda to patient-centered outcomes. (Table X)

Secondary findings included developing new partnerships with key insights, aiding in the development and implementation of research that results in improved therapy, leading to increases in event-free survival, overall survival, and patient/family satisfaction. Several PCORI studies have addressed such issues to ensure all patients have access and make the best decisions for their health [12–18]. 

Our results align with other groups using similar methodologies. Powell et al., employing a similar methodology (Mullins Framework) to engage patients in addressing cancer disparities in Memphis, Tennessee, identified ten research themes [6]. Similarly, Warren et al. developed a research roadmap for dry eye and meibomian gland dysfunction [19]. Wald et al. used the PCORI process to develop a research roadmap for hospital and geriatric medicine [3]. Lastly, two papers from Carlise et al. also reinforce our findings. In the first paper, the authors surveyed adult survivors of adolescent cancer, demonstrating that adolescents wish to participate in the decision-making process and how that involvement would impact their treatment [20]. In the second study, involving parents of newly diagnosed children with cancer, they pilot-tested a “prompt list” to empower parents to ask meaningful questions of their surgeons [21]. 

Based on the results of this study, PSORC has developed and implemented a patient/parent/research evaluation of all PSORC research proposals. In addition, PSORC has prioritized those studies that have a PCOR/CER question. Finally, we have begun work on a question aid for parents.

PCORI was formed to recognize the need for patients and stakeholders to have a greater voice in determining research direction. The roadmap proposed here addresses the necessity for a patient-centered research agenda. We relied heavily on the PCORI framework, starting with inclusive topic generation, followed by systematic review, gap analysis, VOI analysis, and peer review. Other important topics to address include dissemination and evaluation strategy. This methodology will create a rigorously derived and relevant knowledge base for the care of pediatric surgical oncology patients. PSORC has already implemented some strategies emerging from the project. PSORC has an active patient advisory board whose members evaluate all study proposals for PCOR and potential future funding.

Limitations to this process include the inadvertent exclusion of diverse stakeholders, resulting in omitted views and input. Although we reached out to different groups, the participants were a convenience sample. We had 165 responses to the survey; however, determining the denominator from social media is not possible; thus our response rate could be considered low. Additionally, the process does not clearly define how stakeholders should prioritize criteria. For example, is patient-centeredness more important than population impact? Applying a patient-centered approach may prioritize research topics not previously considered critical by pediatric surgeons or policymakers. Lastly, although we developed a research roadmap, funding entities such as PCORI might not find it compelling enough to fund.

In summary, surgical PCOR is lacking and needed to enhance interactions between surgeons and patients/families with pediatric solid tumors. A significant discrepancy in PCOR topics and prioritization exists between pediatric surgeons and families. We employed a mixed-methods, evidence-based approach to identify, understand, and develop research to address these knowledge gaps.

## Data Availability

No datasets were generated or analysed during the current study.
